# Automatic Generation of Connectivity for Large-Scale Neuronal Network Models through Structural Plasticity

**DOI:** 10.3389/fnana.2016.00057

**Published:** 2016-05-26

**Authors:** Sandra Diaz-Pier, Mikaël Naveau, Markus Butz-Ostendorf, Abigail Morrison

**Affiliations:** ^1^Simulation Laboratory Neuroscience – Bernstein Facility for Simulation and Database Technology, Institute for Advanced Simulation, Jülich Aachen Research Alliance, Jülich Research CenterJülich, Germany; ^2^Serine Proteases and Pathophysiology of the Neurovascular Unit, Institut National de la Santé et de la Recherche Médicale UMR-S U919, Caen Normandy University, Groupement d'Intérêt Public (GIP) CYCERONCaen, France; ^3^Institute of Neuroscience and Medicine (INM-6), Computational and Systems Neuroscience, Jülich Research CentreJülich, Germany; ^4^Faculty of Psychology, Institute of Cognitive Neuroscience, Ruhr-University BochumBochum, Germany

**Keywords:** structural plasticity, large scale neural networks, high performance computing, homeostatic growth, self-organizing network

## Abstract

With the emergence of new high performance computation technology in the last decade, the simulation of large scale neural networks which are able to reproduce the behavior and structure of the brain has finally become an achievable target of neuroscience. Due to the number of synaptic connections between neurons and the complexity of biological networks, most contemporary models have manually defined or static connectivity. However, it is expected that modeling the dynamic generation and deletion of the links among neurons, locally and between different regions of the brain, is crucial to unravel important mechanisms associated with learning, memory and healing. Moreover, for many neural circuits that could potentially be modeled, activity data is more readily and reliably available than connectivity data. Thus, a framework that enables networks to wire themselves on the basis of specified activity targets can be of great value in specifying network models where connectivity data is incomplete or has large error margins. To address these issues, in the present work we present an implementation of a model of structural plasticity in the neural network simulator NEST. In this model, synapses consist of two parts, a pre- and a post-synaptic element. Synapses are created and deleted during the execution of the simulation following local homeostatic rules until a mean level of electrical activity is reached in the network. We assess the scalability of the implementation in order to evaluate its potential usage in the self generation of connectivity of large scale networks. We show and discuss the results of simulations on simple two population networks and more complex models of the cortical microcircuit involving 8 populations and 4 layers using the new framework.

## 1. Introduction

Models of large scale neural networks are an important tool for understanding the mechanics of the brain (De Garis et al., 2010; Helias et al., 2012; Eliasmith and Trujillo, 2014). Such models are created based on experimental information that has been collected for years by neuroscientists and combine mathematical methods with algorithms to reproduce observed behavior. It is known that the connectivity of the network plays an essential role in defining the way function is achieved at higher levels of activity. Nevertheless, obtaining accurate measurements of connectivity is complex, even with the most advanced experimental techniques, due to the resolution of sensors and difficult access to the target areas. The dynamics of the connectivity are also not yet well understood, although it has been shown that synaptic plasticity is fundamental for understanding how learning and memory work. Non invasive techniques such as DTI imaging and fMRI scans can provide a glimpse to the real complexity of the problem in structure and function. Higher resolution techniques like electron microscopy (Gray, 1959), photostimulation (Dantzker and Callaway, 2000) and electrophysiological recordings (Thomson et al., 2002) provide more detailed connectivity information of specific regions. Regardless, creating an exact connectivity map of even a small region of the brain is extremely challenging (Deco et al., 2008; Essen et al., 2012; Van Essen and Ugurbil, 2012; Reckfort et al., 2013). This poses a significant problem for the modeling approach, as connectivity must be specified. For small networks, parameter scans can be carried out with respect to the unknown or imprecisely known connection probabilities between populations. For larger networks, which are more costly to simulate and also potentially have many more unknown connectivity parameters, this approach is hardly feasible.

One way to address the issue of modeling connectivity within a neural network is to allow a network model to determine its own suitable connectivity to achieve target activity patterns, e.g., experimental measurements of the spiking frequency, which is easier to measure accurately than connectivity. In addition to addressing the problem of network model specification, a framework that accounts for the appearance and disappearance of synapses on the basis of network activity can provide insight into how connectivity is generated during development and learning or even on how healing after lesions takes place (De Paola et al., 2006). It can also help understand how certain structures arise as a result of exposition to adequate external stimuli during critical periods in the development of the brain (Hensch, 2005) and the mechanisms underlying experience dependent structural synaptic plasticity (Holtmaat and Svoboda, 2009).

An appropriate model of structural plasticity that incorporates the dynamic generation, deletion and rewiring of synapses within a network was presented by Butz and van Ooyen (2013). In this model, synapses are represented as connections between pre and a post synaptic elements. The growth or diminishment of these synaptic elements is an independent process for each neuron. The model is based on the idea that plasticity in cortical networks is mainly driven by the need of individual neurons to homeostatically maintain their average electrical activity. As a consequence, if activity is lower than a desired set-point, neurons will form synaptic elements, and remove them when activity becomes too high. Additionally, a minimum level of activity is needed to form synaptic elements at all. If activity falls below this level the neuron will remove synaptic elements, too. Results show that small networks of hundreds or thousands of neurons robustly grow toward a stable homeostatic equilibrium of activity and connectivity. An important advance on earlier work is that all cell types had different desired average firing rates (achieved by different homeostatic set-points) and developed connectivity accordingly. It was shown that these local rules for structural plasticity can account for network rewiring after a partial loss of external input (deafferentation) and shows remarkable similarities with biological data from network rewiring in the primary visual cortex after focal retinal lesions (Keck et al., 2008; Yamahachi et al., 2009). Further analysis by Butz et al. (2014) of changes in network topology revealed that betweenness centrality could be used as an indicator of successful brain repair, in the sense that it is related to the ability of the neurons to restore their electrical activity by network rewiring. It was concluded by the authors that structural plasticity may account for network reorganization on different spatial scales.

In this work, we provide a complete description of how the structural plasticity model proposed by Butz and van Ooyen (2013) could be implemented in the neuronal network simulator NEST (Gewaltig and Diesmann, 2007) in order to create self-organizing large scale neural networks. We evaluate the scalability of the implementation and assess the performance of the model on two use cases. We demonstrate that our implementation is capable of self-organizing the connectivity within a cortical microcircuit model consisting of 100, 000 neurons in total, starting with a fully disconnected setup. We also show the scenario where partial information of the connectivity is given as initial condition and an stable connectivity pattern is obtained in the end.

The structural plasticity extension to NEST is included in release 2.10.0 (Bos et al., 2015) and creates a novel possibility for setting up large-scale neuronal networks. While supercomputers are required for very large-scale simulation, we show that smaller networks can also be run on a personal workstation or laptop according to the NEST development philosophy. This is a fundamental advantage of this implementation of structural plasticity in terms of capacity to test different configurations, as it provides high flexibility and portability for the neuroscientist.

The corresponding extension of the Python interface of NEST (PyNEST) allows the user to set up their own structural plasticity experiments for large scale networks.

The rest of this work is divided into three major parts. The first describes the major elements of the structural plasticity algorithm and the set of tests that were designed in order to measure the performance of an implementation of this algorithm. We also present some use cases for the structural plasticity framework. In the second part we provide the results of the technical implementation in NEST and describe how the design matches the memory and speed requirements for large scale simulations. We also present results for the use cases described in the previous section. In the third part, we discuss the results of the implementation and performance tests.

Some of this material has previously been presented in abstract form (Naveau and Butz, 2014).

## 2. Materials and methods

### 2.1. The algorithm of structural plasticity

The original formulation of the structural plasticity algorithm defined in Butz and van Ooyen (2013) consists of three repeating steps which are described as follows:

1. Update in electrical activity and intracellular calcium concentration. The electrical activity is calculated for each neuron on a millisecond timescale. The time-averaged level of the neuron's electrical activity drives changes in neuronal morphology. Intracellular calcium concentration is updated according to the electrical activity as follows:
  (1) $$\frac{d\text{Ca}}{dt}=\{\begin{array}{ll} -\frac{Ca(t)}{\tau }+\beta & \text{if the neuron fires}\\ -\frac{Ca(t)}{\tau } & \text{otherwise} \end{array}$$
  where τ is the calcium decay constant and β is the calcium intake constant which indicates how much calcium is accumulated each time the neuron fires. Calcium concentration is linearly proportional to average firing rate and thus is the measure that is used to guide the growth dynamics of the synaptic elements.
2. Update in synaptic elements. The detailed morphology of the synaptic elements is abstracted, and is represented in this formulation only by the number of possible synaptic contacts on axons (axonal elements representing axonal boutons: senders of synaptic activity) and on dendrites (dendritic elements representing dendritic spines: receivers of synaptic activity) collectively called synaptic elements. Synaptic elements are created or deleted according to a homeostatic rule. In general, the homeostatic rule will create synaptic elements when the activity is lower than the desired setpoint and delete them when the activity is higher until the desired activity level is achieved. This homeostasis is represented by a curve which defines how quickly new elements are created or deleted according to the current level of electrical activity. The original work considers two types of growth curve, linear and Gaussian:
  **Linear:**
  $$\frac{dz}{dt}=\nu (1-\frac{1}{\epsilon }Ca(t))$$
  where ν is the growth rate and ϵ is the target level of calcium concentration that the neuron should achieve.
  **Gaussian:**
  $$\frac{dz}{dt}=\nu \left(2\exp\left(-\frac{Ca(t)-\xi }{\zeta }\right)-1\right)$$
  where ξ = (η+ϵ)∕2, $\zeta =\left(\epsilon -\eta \right)∕2\sqrt{\ln2}$ and ν is the growth rate as before. In this Gaussian growth curve, η represents the minimum amount of calcium concentration that the neuron must have in order to start creating new synaptic elements. Same as in the linear growth, ϵ represents the target level of calcium concentration that the neuron should achieve.
  A synaptic element is formed (or deleted) when the rounded down *z* value increase (or decrease) by one. Newly-formed synaptic elements are initially vacant and available for synapse formation.
3. Update in connectivity. In every connectivity update, available synaptic elements allow the formation of new synapses and deleted synaptic elements dictate synapse breaking. Every available synaptic element has the same probability to be randomly chosen for a new connection. Synaptic elements to be deleted are also chosen in a uniformly random manner out from the pool of already connected elements. It is important to notice that in this algorithm when a synapse breaks due to the deletion of one synaptic element, the counterpart remains and becomes vacant again. This remaining counterpart can form a new synapse at the next update in connectivity. This effect models network rewiring by re-routing of axons or dendrites.

An important characteristic of this algorithm, is that it relies on global communication to update the connectivity in the network, as available compatible synaptic elements must be matched during the simulation to create new connections. This must be taken into consideration for the design of any implementation of this model.

### 2.2. Scalability

To assess the scalability of the framework, we designed strong and weak scaling tests of the structural plasticity implementation. For all tests, networks with 80% excitatory and 20% inhibitory neurons were created. The growth rate for synaptic elements in the simulation was set to 4.0 × 10^−4^elements∕ms for the excitatory elements of the inhibitory population and 1.0 × 10^−4^elements∕ms for all the other elements. The set point for desired calcium concentration in the excitatory population was defined as 0.05 Ca^2+^, while in the inhibitory population it was set to 0.2 Ca^2+^. The calcium concentration intake constant was set to β = 0.001 and the calcium concentration decay constant to τ = 10000.0 for all neurons. The post synaptic amplitude of individual synapses was set to 1.0 mV. External input was provided using a Poisson generator with a frequency of 10^4^ Hz. The post synaptic amplitude of individual synaptic input was set to 0.01 mV. The simulation was run for 100 s, with a step size for the numerical integration of 0.1 ms. The updates in the network connectivity were performed every 10 ms. These values were chosen as they proved to be one parameter combination that allowed for stable self-organizing growth of the network toward the homeostatic equilibrium (See Section 3.3.1 for additional comments on the selection of this parameter set).

Weak scaling tests were performed for networks with 5000 neurons per node and settings of 1, 2, 4, 8, and 16 nodes, each node using 28 cores. Strong scaling tests were performed with a network of 100, 000 on the same hardware configurations as the weak scaling tests. Only physical cores were used, no simultaneous multithreading was enabled. A hybrid optimization approach was chosen, in which MPI is used for communication between nodes and OpenMP for intra node communication. All measurements were performed on the JUROPATEST cluster, which provides up to 70 nodes (T-Platforms V210s Blades), each with 2 × Intel(R) Xeon(R) CPU E5−2695 v3 (Haswell) with 14-core processors (2.30 GHz) and 128 GB DDR memory, running with Scientific Linux release 6.5 (Carbon).

### 2.3. Use cases for the structural plasticity framework

The main objective of the structural plasticity framework is to provide the user with a tool to model the dynamic creation and deletion of synapses between neurons of a neural network in a scalable manner. There are several applications in which structural plasticity can be used. In this section we detail two use cases as examples. The first use case shows the basic functionality of the framework and how it can be used to study the relationship between connectivity and activity. We also show how this simple set-up can model critical development periods in the network connectivity. The second example is a more complicated case with several populations, where the objective is to show how connectivity can be self-generated in a network by using the synaptic element growth curves as connectivity fitness rules. All simulations were carried out with NEST version 2.8.0 extended by our structural plasticity implementation.

#### 2.3.1. A simple two population network

In this initial use case, we generate a network with a total of 1000 leaky integrate and fire neurons, 80% excitatory and 20% inhibitory. For the excitatory neurons, η = 0.0, ϵ = 0.05 and ν = 1.0 × 10^−4^ elements∕ms. For the inhibitory neurons, η = 0.0, ϵ = 0.2 and ν = 1.0 × 10^−4^elements∕ms, except for the excitatory elements which had ν = 4.0 × 10^−4^elements∕ms. The connectivity in the system was allowed to evolve using a Gaussian growth curve for 3000 s, with an integration step of 0.1 ms and a delay of the connectivity update equal to 100 integration steps. The simulations were performed on a workstation with 8 Intel core i7 − 4770@3.4 GHz CPUs running openSUSE 13.1.

#### 2.3.2. The cortical microcircuit network

**Figure 1:**
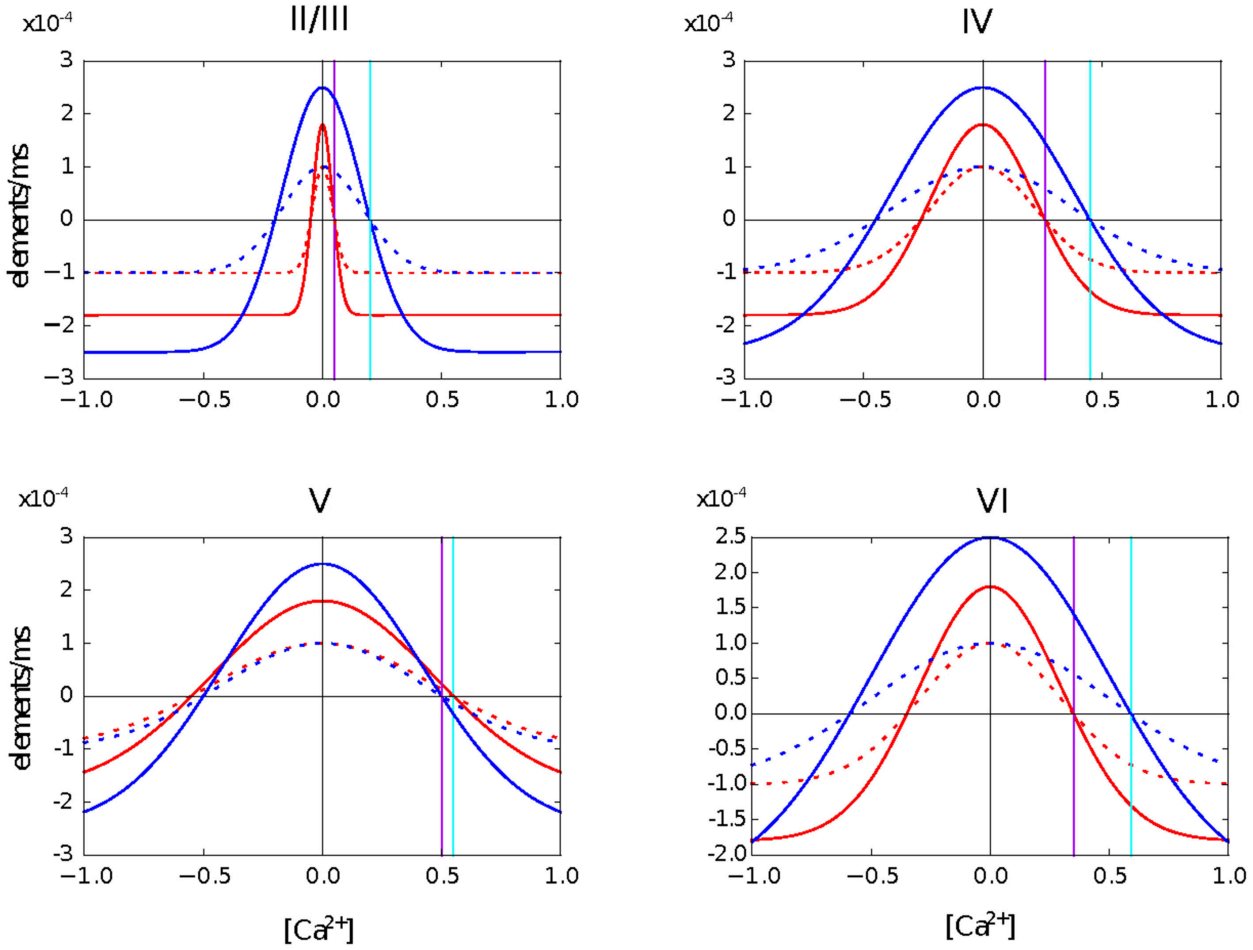
**Growth curves for each synaptic element in each layer of the cortical microcircuit model**. The growth curves define the rate at which synaptic elements are created depending on the amount of calcium concentration in the cell at the moment. Red curves are for neurons in the excitatory population. Blue curves are for neurons in the inhibitory population. Solid lines are for the excitatory synaptic elements and dotted lines represent inhibitory synaptic elements. The vertical purple line defines the target level of calcium concentration for excitatory neurons and the vertical cyan line represents the target level of calcium concentration for inhibitory neurons. It is important to highlight that all synaptic elements of the same neuron must have a growth curve with the same target level of calcium concentration, otherwise equilibrium will never be reached.

In this second use case, we create a four layer network based on the model of the cortical microcircuit proposed by Potjans and Diesmann (2014). Each layer contains one inhibitory and one excitatory population of leaky integrate and fire neurons. In the simulations presented here, the network starts with the same number of neurons in each population as in the previous study, but without any synaptic connections. For each population, we define a level of desired mean electrical activity based on experimental literature and a growth curve which defines the dynamics of the variation in the number of pre- and post-synaptic elements. These are Gaussian shaped curves with two intersections with the x-axis that determine the minimum amount of electrical activity required to form any synapse (η), and the target mean calcium concentration for the neuron (ϵ). The curves are illustrated in Figure 1.

In the first example, we tune the growth rate to achieve an stable growth regime for the network connectivity. This means that the structural plasticity algorithm will stop creating and deleting synaptic connections when the desired mean activity is reached, and that this mean activity is actually reached on average in each population. In a second example, the growth rate provided leads to an unstable connectivity pattern, where the target mean electrical activities are never reached by all populations. A table containing the parameters for both cases can be seen in Appendix in Supplementary Material.

A third example was run to illustrate a more common situation where there are some assumption about the connectivity in a network and where the structural plasticity framework can help to find a suitable balance between excitation and inhibition in the network. Here we used the original model of Potjans and Diesmann (2014) and enable the structural plasticity after an initial stabilization period of 30 s.

Simulations were performed on JUROPATEST (70 nodes with 2 × 14-core processors Intel(R) Xeon(R) CPU E5−2695 v3 (Haswell) at 2.30 GHz and 128 GB DDR memory, running with Scientific Linux release 6.5) and JURECA (with 260 compute nodes with Intel Xeon E5−2680 v3 Haswell CPUs with 2 × 12 cores per CPU, 128 GB of RAM per node and running on CentOS 7 Linux distribution).

## 3. Results

### 3.1. Implementation of the structural plasticity model into NEST

**Figure 2:**
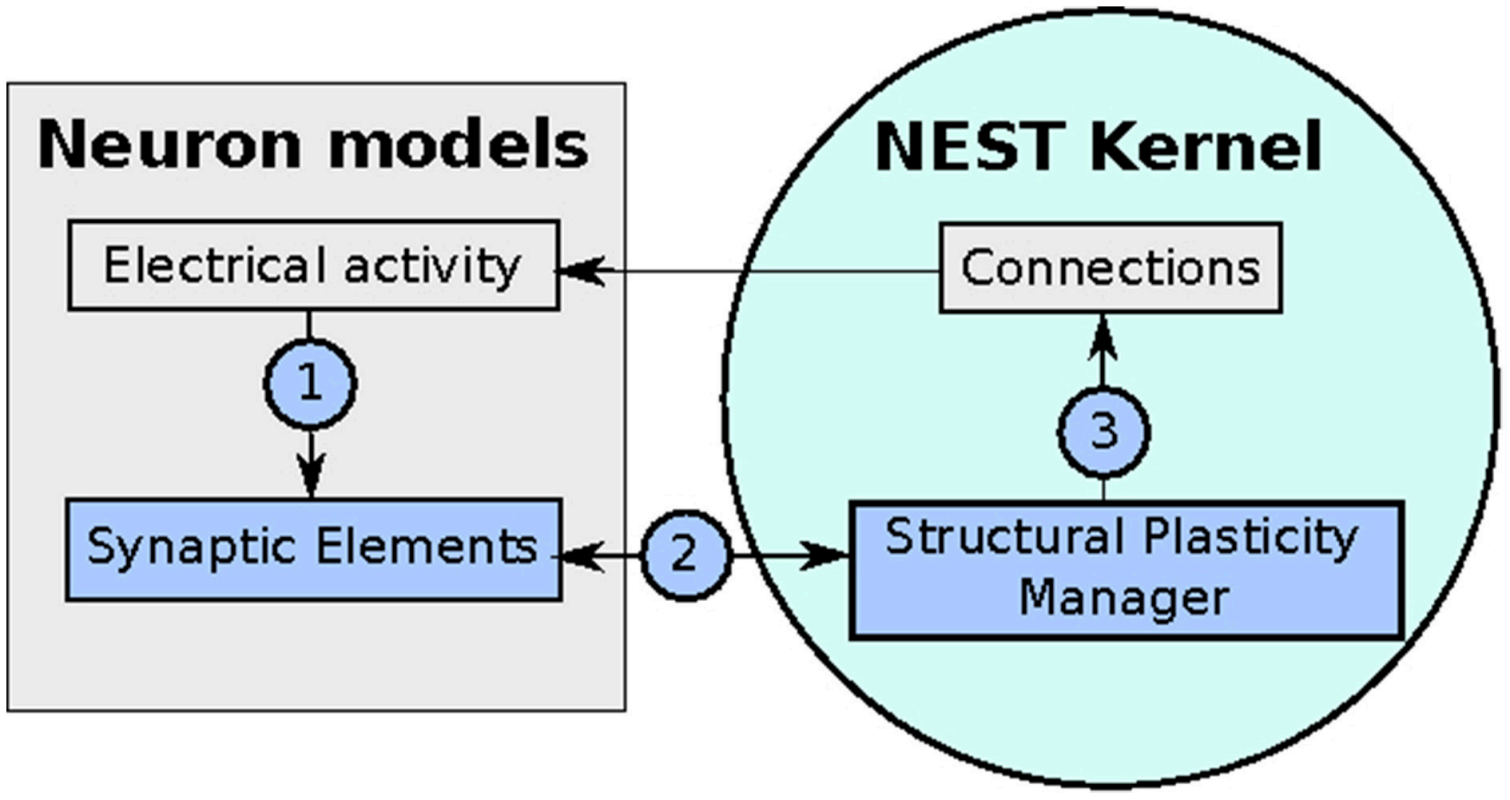
**Diagram of the implementation of the structural plasticity model in NEST**. In (1) the number of synaptic elements is calculated depending on the electrical activity of the neuron. These calculations are optimized using MPI and OpenMP. In (2) the structural plasticity manager gathers the number of synaptic elements per neuron using MPI directives and in (3) creates or deletes synapses to update the connections between neurons using MPI and OpenMP.

**Figure 3:**
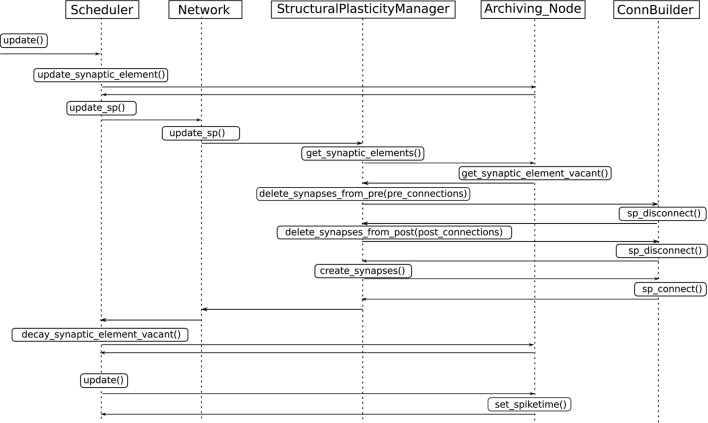
**Integration of the new structural plasticity calls into the normal simulation flow of NEST**.

The implementation of the structural plasticity algorithm described in this work is based on the version 2.8 of the NEST software Eppler et al. (2015). In accordance with the original formalization described in Section 2.1, the algorithm consists of three repeating parts which can be visualized in a general form in Figure 2 and described as follows:

1. Update in electrical activity and intracellular calcium concentration. The *Archiving_Node* class, which is the general interface for all neurons, was modified and new variables to store the of the intracellular calcium concentration, the calcium decay and calcium intake constants were added. The *Archiving_Node::set_spiketime* method was also modified to update the calcium concentration according to the first case defined in (1), which is performed at every time the neuron spikes. The Node class was modified by adding the method *Archiving_Node::update_synaptic_element*. This method updates the calcium concentration according to the second case defined in (1). This method is called by the *Scheduler* class when every synaptic update interval is reached.
2. Update in synaptic elements. The first step taken in order to design and develop a framework for synaptic elements (e.g., axonal boutons and dendritic spines) was to redefine synapses in such a way that they can now be described using connection elements. This description can be applied to every available neuron model in NEST for generating electrical activity. The design also considers that the users can define their own synaptic elements and their corresponding growth dynamics. The class *SynapticElement* was created in order to represent the connection points for the neurons. The class *GrowthCurve* was also created in order to define the homeostatic rules which guide the creation and deletion of synaptic elements. Currently, the available growth dynamics are based on either a linear or a Gaussian growth curve. The linear growth curve uses an exact integration method to update the number of synaptic elements, while the Gaussian growth curve uses a forward Euler integration method. The framework can be further extended by the user to incorporate more complex element growth dynamics models. An example of such curves is shown in Figure 1, where independent dynamics for each type of element in a network of 8 populations (see use case on cortical microcircuit) have been defined. Synaptic elements are used as a discrete value, the actual number of available synaptic elements is an integer truncated from the float variable used to represent them. The *Archiving_Node* class now incorporates a map data structure to store the synaptic elements. The method *Archiving_Node::update_synaptic_element* takes care of updating the number of each of the synaptic elements in the map using the value of the calcium concentration at the time of the call and the corresponding growth curve. The method *Archiving_Node::decay_synaptic_element_vacant* takes care of deleting a percentage of the unused or vacant synaptic elements on every call. Both methods are called by the *Scheduler* at the end of every synapse update interval.
3. Update in connectivity. To coordinate the changes in the structure of the network, a new *StructuralPlasticityManager* class was implemented. At the end of every synapse update interval, the *Scheduler* calls the the newly implemented structural plasticity connectivity manager via the *Network* class. The *StructuralPlasticityManager* determines, for each neuron, how many vacant synaptic elements are available for new synapse formation and how many deleted synaptic elements caused synapse breaking. Then it makes use of the *ConnBuilder* in order to create or delete connections. For this, the *ConnBuilder* was extended to include the new methods *ConnBuilder::sp_disconnect_* and *ConnBuilder::sp_connect_*. Once new synapses are formed, synaptic elements are tagged from “vacant” to “connected.” It is important to notice that when a synapse breaks due to the deletion of one synaptic element, the counterpart remains and becomes vacant again. This remaining counterpart can form a new synapse at the next update in connectivity. This effect preserves the network rewiring capabilities of the original formulation. A detailed diagram of how the new calls are integrated into the normal simulation flow of NEST can be seen in Figure 3.

An important feature that we implemented to simulate structural plasticity in NEST is the ability to create and delete synapses during the simulation time. Our new implementation of the connection management overcomes the limitation of the NEST simulator that currently models networks with a fixed connectivity. We have implemented the dynamic creation and deletion of synapses using the new connection framework released in version 2.6.0. The new connection framework improves memory usage to store connection data and reduces the computation time needed to create a connection.

The main limitation of the structural plasticity algorithm described by Butz and van Ooyen (2013) is that it requires global knowledge of the synaptic elements of the entire network. Fortunately, the MPI global communications, also used by the NEST kernel to communicate the electrical activity between the neurons during the simulation, do not pose a substantial bottleneck since changes in connectivity are assumed to take place on average around a factor of 100 times slower than changes in electrical activity. Therefore selecting a biologically realistic growth rate of around 10^−4^elements∕ms will result in an exchange of data that is sufficiently low rate so as not to impact the scalability of the simulator as a whole. At the end of each connectivity update step, the number of created/deleted synaptic elements per neuron are communicated to all MPI processes and a global shuffle subsequently assigns the new pairs of neurons that should be connected, and likewise chooses existing connections for deletion. In the current implementation, no topological constraints are taken into account while deciding which neurons will be connected. The probability of two neurons connecting to each other depends solely on the number of available compatible synaptic elements between them. The actual creation and deletion of the synapses is finally done in parallel using the NEST connection framework. As stated before, a single update in connectivity should not produce a major modification of the network. That means that only a small part of the neurons should create or delete a synaptic element between two updates in connectivity.

It is important to highlight that the usage of global communication is a characteristic of the technical implementation of the algorithm and is not related to the functionality of the model. If topology was to be taken into account, the ability of a neuron to connect to any other would be limited by the constraints imposed by its relative position to others. Global communication would still be used by the implementation, but only relevant information would be taken into account to define the connectivity. The local homeostatic rules only define the creation or deletion of synaptic elements per neuron. The number of available synaptic elements is transmitted globaly and the synaptic plasticity manager takes care of forming new synapses or deleting existing ones based on this information.

The update of electrical activity and of the number of synaptic elements is performed by every individual neuron and therefore benefits from the parallel framework already implemented in NEST. Indeed, the NEST software has already demonstrated its high scaling properties on supercomputer, including the JUQUEEN system (Helias et al., 2012; Kunkel et al., 2014).

Finally, the Python interface of NEST (PyNEST) was extended to allow users to easily set up the structural plasticity parameters. It is important to highlight that the user can enable structural plasticity inside the simulation and then disable it when the network has achieved a desired connectivity pattern or activity level. The user can now also delete synapses even without enabling structural plasticity, in a similar way as the connect functions work in NEST.

#### 3.1.1. Setting up a network in NEST with structural plasticity

In this section we will introduce the high level functions that are introduced into NEST with the structural plasticity framework using PyNEST.

In order to set up the network using structural plasticity, one first needs to define the time at which updates in the structure of the network should take place as follows:


 
  nest.SetStructuralPlasticityStatus({
  
  'structural_plasticity_update_
             interval':
  
          update_interval,
 })


The next step is to define the synapses which can be dynamically modified by the structural plasticity manager during the simulation. This is achieved by:


 
  nest.SetStructuralPlasticityStatus({
  
    'structural_plasticity_synapses': {
  
  'structural_plasticity_synapse_
           ex': {
  
  'model': 'structural_
          plasticity_synapse_ex',
  'post_synaptic_element'
             : 'Den_ex',
  'pre_synaptic_element'
              : 'Axon_ex',
 
},
'structural_plasticity_synapse_
   in': {
 
  'model': 'structural_
           plasticity_synapse_in',
  'post_synaptic_element'
                : 'Den_in',
  'pre_synaptic_element'
                 : 'Axon_in',
 
    },
  }
})


Here, two types of synapses are being defined, one for the excitatory synapses and another one for the inhibitory synapses. It is important to notice that in this definition, a name for the post and pre synaptic elements is also specified. This allows the structural plasticity manager to create new synapses of the type specified in model when synaptic elements related to this label become available. This way of setting up the dynamic synapses also allows the user to define static connectivity constraints in the network. This can be achieved by using one synapse model which is not registered for structural plasticity to define this fixed connectivity. For the moment, no other constraints in connectivity like indegree or outdegree ranges can be specified. Nevertheless, thanks to its flexible design, the model can be extended to add new constraints.

Next step involves defining the growth curves for the synaptic elements defined above. This is done as follows:


 
growth_curve_e_e = {
  
  'growth_curve': “gaussian”,
  'growth_rate': 0.0001,
  'continuous': False,
  'eta': 0.0,
  'eps': 0.05,
}


This is an example of a Gaussian growth curve where the minimum level of calcium concentration required to start generating synaptic elements is η = 0.0 Ca^2+^, and the desired calcium concentration is set to ϵ = 0.5 Ca^2+^. Finally, the rate at which the synaptic elements will grow is ν = 1 × 10^−4^elements/ms. Independent growth curves can be created for each synaptic element.

Now that we have defined the growth curve, we can assign this growth curve to the synaptic elements that each neuron will be able to grow. After that, we create the neurons and let NEST know that these synaptic elements are linked to the neurons:


 
synaptic_elements = {
  
      'Den_ex': growth_curve_e_e,
      'Den_in': growth_curve_e_i,
      'Axon_ex': growth_curve_e_e,
}
nodes = nest.Create('iaf_neuron',
      number_excitatory_neurons)
nest.SetStatus(nodes, 'synaptic_
      elements', synaptic_elements)


In this case we are creating the neurons pertaining to the excitatory population. Each neuron has three types of synaptic elements, one dendritic excitatory, one dendritic inhibitory and one axonal excitatory.

The final step is to enable structural plasticity and simulate:


  nest.EnableStructuralPlasticity()
  nest.Simulate(t_sim)


A complete PyNEST example which describes how to create a network with two populations, enable structural plasticity and simulate the network is available as Supplementary Material for this paper.

### 3.2. Scalability

While the update in electrical activity has been proven to scale up to 10^9^ neurons, it is important to verify that updating the number of elements and the deletion and formation of synapses does not restrict the expected scaling, at least in the desired regime of up to 10^6^ neurons. Updates in synaptic elements and connectivity make use of MPI's “AllGather” communication scheme to communicate the data. This collective communication is also used by the NEST kernel to communicate the spiking activity between the neurons during the simulation. Although AllGather implements communication between all processes, it is very unlikely that a huge amount of data has to be communicated when a reasonable growth rate of around 10^−4^elements∕ms because updating the number of synaptic elements and the connectivity are very slow processes compared to the update in electrical activity.

#### 3.2.1. Weak scaling

**Figure 4:**
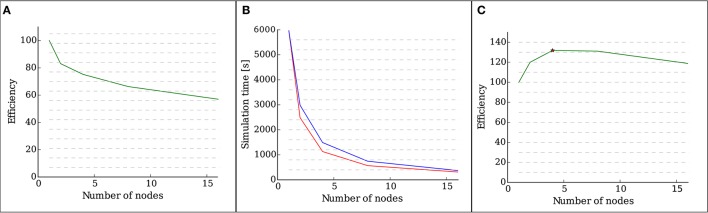
**Results of the scalability tests performed with structural plasticity**. **(A)** Efficiency as a function of the number of nodes for 5000 neurons in the weak scaling test. The network was allowed to grow synapses following the structural plasticity rules during a simulation of 100 s of biological time. **(B)** Simulation time (red curve) as a function of the number of nodes for a network of 100, 000 neurons and in the strong scaling test. The blue curve indicates ideal linear scaling. **(C)** Efficiency as a function of the number of nodes for a network of 100, 000 neurons in the strong scaling test. The peak scaling efficiency is marked with a star.

Figure 4A shows the efficiency, defined as the speed-up divided by the number of nodes, of the implementation as measured by a weak scaling test with 28 OMP threads running on each node. It is visible that, as the number of neurons increases, so does the total number of synapses. The presence of new synapses leads to an increase of communication between neurons, which leads to a decrease in the efficiency of the simulation.

#### 3.2.2. Strong scaling for a network of 100,000 neurons

Figure 4B shows the computation times of the strong scaling tests for a network of 100, 000 neurons, and Figure 4C shows the efficiency, defined as speed-up divided by the number of nodes, of the strong scaling test. The peak efficiency is achieved with 4 nodes and 112 cores. These results show supra-linear scaling for this network. In Morrison et al. (2005) and Plesser et al. (2007), supra linear scaling for biological neural networks on NEST was demonstrated due to increasingly efficient caching.

These results show that the introduction of the new structural plasticity framework into NEST has no impact in the scalability of the simulation up to a network size close to that of a cortical column if a suitably low growth rate is selected.

### 3.3. Performance on the use cases

#### 3.3.1. A simple two population network

**Figure 5:**
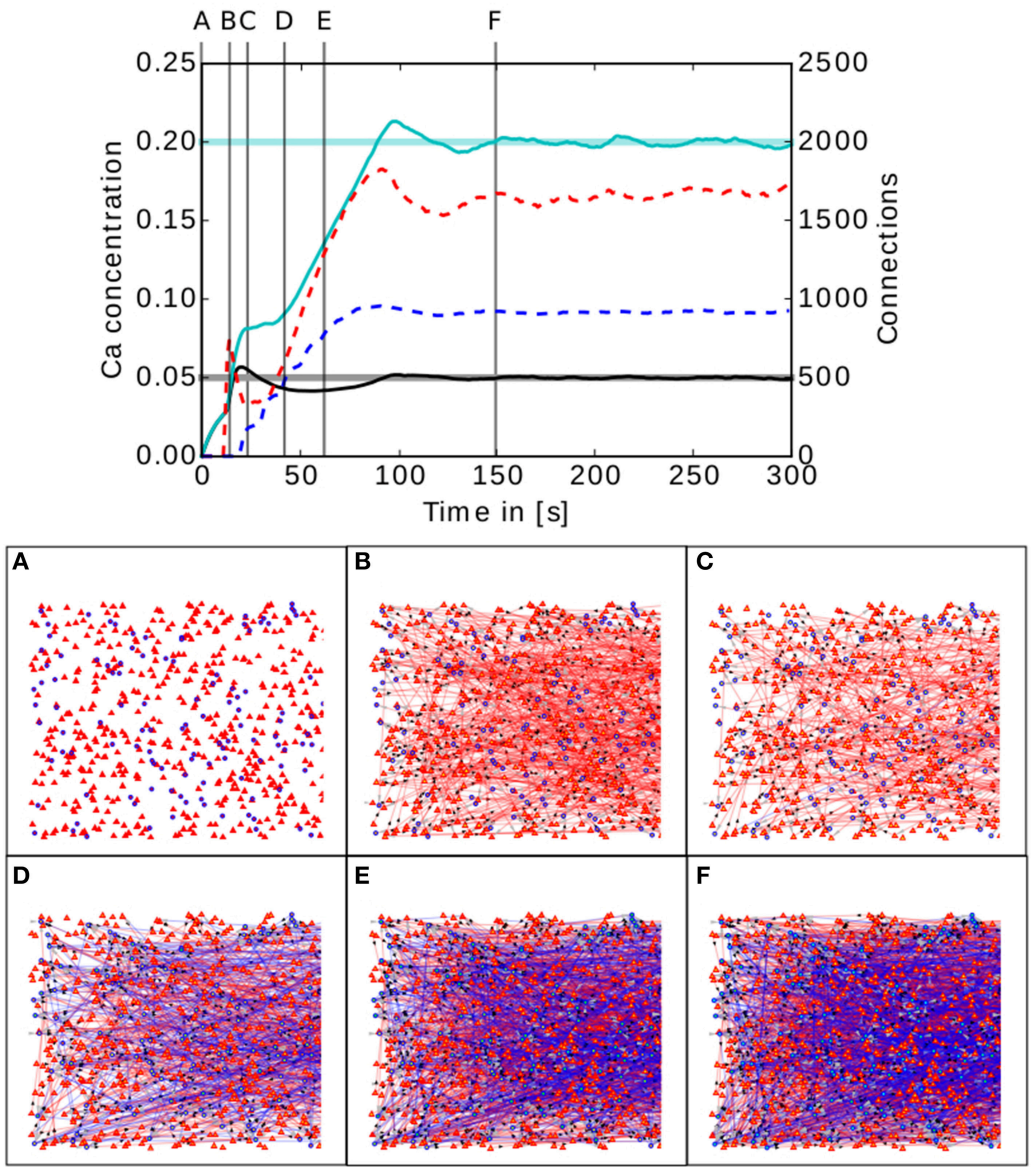
**Upper panel: Calcium concentration and numbers of connections as functions of time in a simple two population network**. The cyan and black curves show the calcium concentration measured in the inhibitory and excitatory populations, respectively. The paler horizontal lines indicate the corresponding target levels ϵ. The blue and red dashed curves indicate the total number of connections in the inhibitory and excitatory populations, respectively. Vertical gray lines indicate the times of the snapshots displayed in the lower panel. **Lower panel (A–F)**: Evolution of the connectivity in the two population network visualized using MSPViz. Images show half of the total amount of neurons in the network, where triangles represent excitatory neurons and circles inhibitory neurons. Red lines indicate excitatory connections while blue lines indicate inhibitory connections.

The upper panel of Figure 5 shows the evolution of the calcium concentration and total number of connections for the two population model described in 2.3.1.

The lower panel of Figure 5 shows a graphical representation of the evolution of the connectivity in the network. During the first 30 s of the simulation, mostly excitatory connections are created (Figure 5A). This allows the calcium concentration to increase in both populations. When the target mean electrical activity is reached and overshoots in the excitatory population (Figure 5B), the number of excitatory connections starts to decrease (Figure 5C) until the desired level of calcium concentration is achieved and stabilized in the excitatory population. However, both pre- and post-synaptic elements in the inhibitory population are still being created because it has not yet reached its target mean electrical activity. It is important to remember that neurons have no information regarding the global status of the network and the evolution of their synaptic elements depends solely on the predefined homeostatic local rules. At around 40 s (Figure 5D), an increment in excitatory connections is triggered by the enhanced levels of inhibition. This leads to a complete rewiring of the network (Figure 5E). The trend is preserved until the mean electrical activity in the inhibitory population is also reached (Figure 5F).

In this network setting, the inhibitory population has a higher level of activity than the excitatory population. It is important to remember that the probability of two neurons connecting depends only on the number of available compatible synaptic elements between them. At the start of the simulation, the inhibitory population must offer more post-synaptic elements for excitatory synapses than the excitatory population, otherwise the excitatory population would reach equilibrium first and cease to create excitatory pre-synaptic elements. As a result, not enough excitatory synapses would be created to the inhibitory population and it would never reach the desired level of activity. It is important to remember that the structural plasticity parameter space is broad and a certain amount of exploration is required to discover combinations for each synaptic element which take the network to equilibrium. However, there is in general no unique combination of parameters leading to equilibrium, and different equilibrium combinations will typically produce different connectivity patterns. At this point, biological constraints must be applied to choose between them.

#### 3.3.2. The cortical microcircuit network

**Figure 6:**
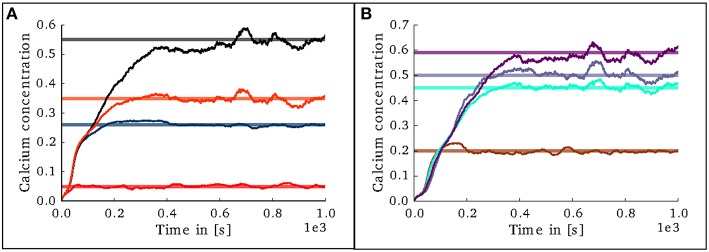
**Evolution of calcium concentration in each layer of the cortical microcircuit model**. Pale horizontal lines indicate the target concentration of the corresponding population. **(A)** Excitatory populations in layers II/III (red), IV (blue), V (black), and VI (orange). **(B)** Inhibitory populations in layers II/III (brown), IV (cyan), V (gray,) and VI (purple).

**Figure 7:**
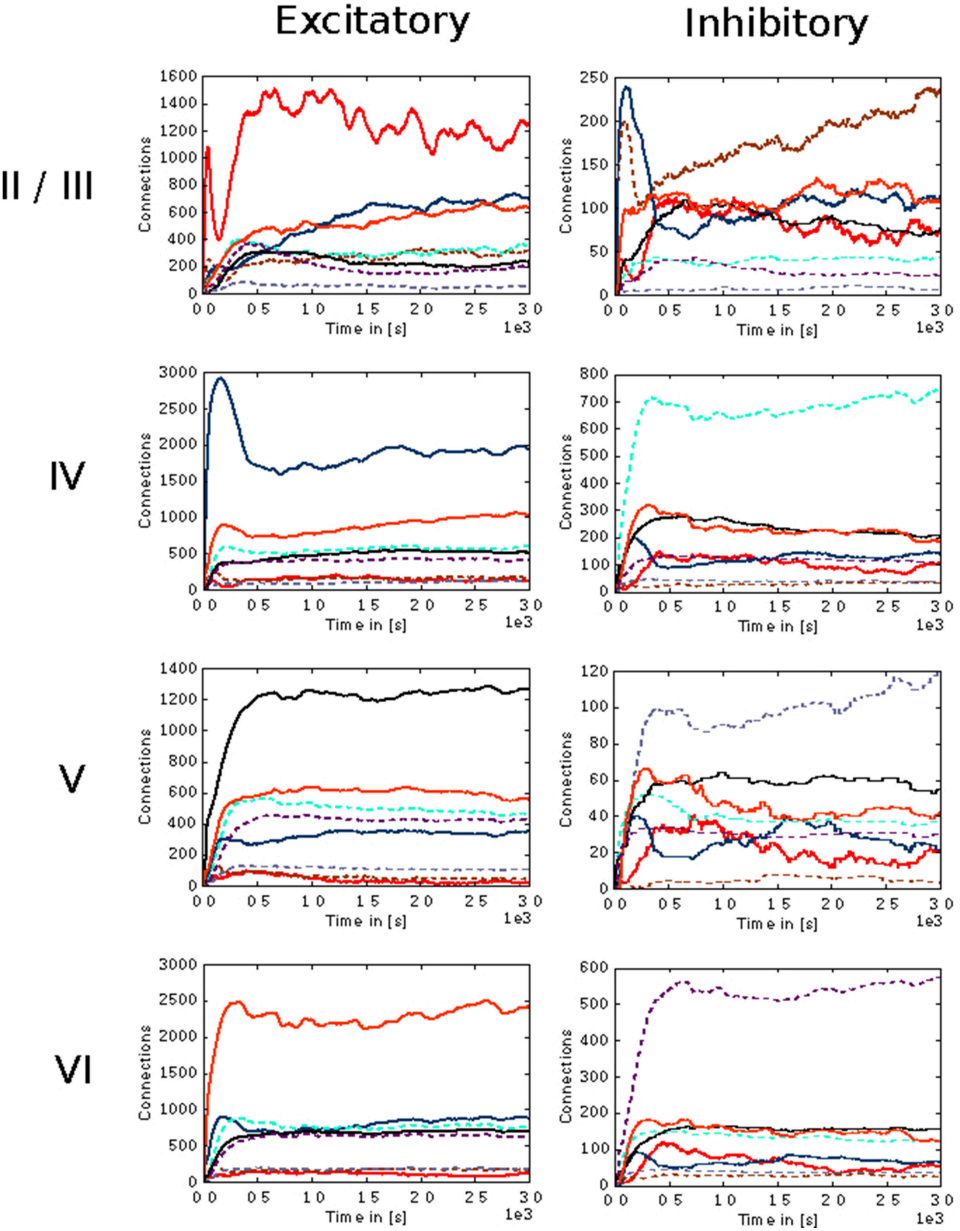
**Evolution of connectivity in the microcircuit model resolved by source and target population**. Panels on the left and right illustrate efferent connections from excitatory and inhibitory populations, respectively, while the vertical arrangement indicates the layer of the source neurons. In each panel, the numbers of connections to each of the eight population in the model are shown as a function of time. The color of the curves indicates the target population, as in Figure 6: connections to excitatory populations in layers II/III (red), IV (blue), V (black), and VI (orange) are shown as solid curves, and connections to inhibitory populations in layers II/III (brown), IV (cyan), V (gray), and VI (purple) are shown as dashed curves.

In the case of the cortical microcircuit model described in 2.3.2, Figure 6 shows the changes in calcium concentration, while Figure 7 shows the evolution of connectivity among layers as the simulation runs. In this case, parameters which lead to stable network connectivity were chosen. Reaching stable connectivity in the networks takes around 700 biological seconds of simulation, which takes 24 h using 25 nodes and 24 cores per node in the JURECA cluster to simulate. It is visible that during the first 20–30 s of simulation, connectivity highly increases on every layer. After the initial overshoot, a smoother approximation toward the desired activity levels is achieved. As seen only from the calcium concentration diagram, the evolution of the network appears to be quite stable. Regardless, the connectivity plots show a continuous dynamical reorganization. While neurons on some layers might start deleting connections due to excess of activity, the post-synaptic neurons must then create new connections in order to compensate for missing activity in case they have not reached their setpoint yet. This leads to a continuous search for compensating excitation and inhibition which must satisfy the requirements of all 8 populations. From Figure 7 it can be seen that outgoing connections from excitatory populations on layers IV, V, and VI are quite stable. On the other hand, layer II/III exhibits the highest amount of reorganization, both from the excitatory and inhibitory populations. This might be due to the fact that their reduced target levels of activity might be easily influenced by variations in all other layers. Inhibitory populations on all layers in general exhibit a higher degree of reorganization during the whole simulation.

The search space of connectivity parameters for this model of the cortical microcircuit is large as each setup requires 64 values to be defined. If a brute force exploration would be performed on these parameters by simulating each combination for 1 biological second, only 1−2 values per parameter could be considered before more biological seconds would be simulated than using the structural plasticity approach. When adequate synaptic element growth curves are defined, the structural plasticity framework allows a progressive exploration of the space in which the dynamics of the the 8 populations are balanced at every step, thus providing an efficient way to find stable connectivity combinations.

**Figure 8:**
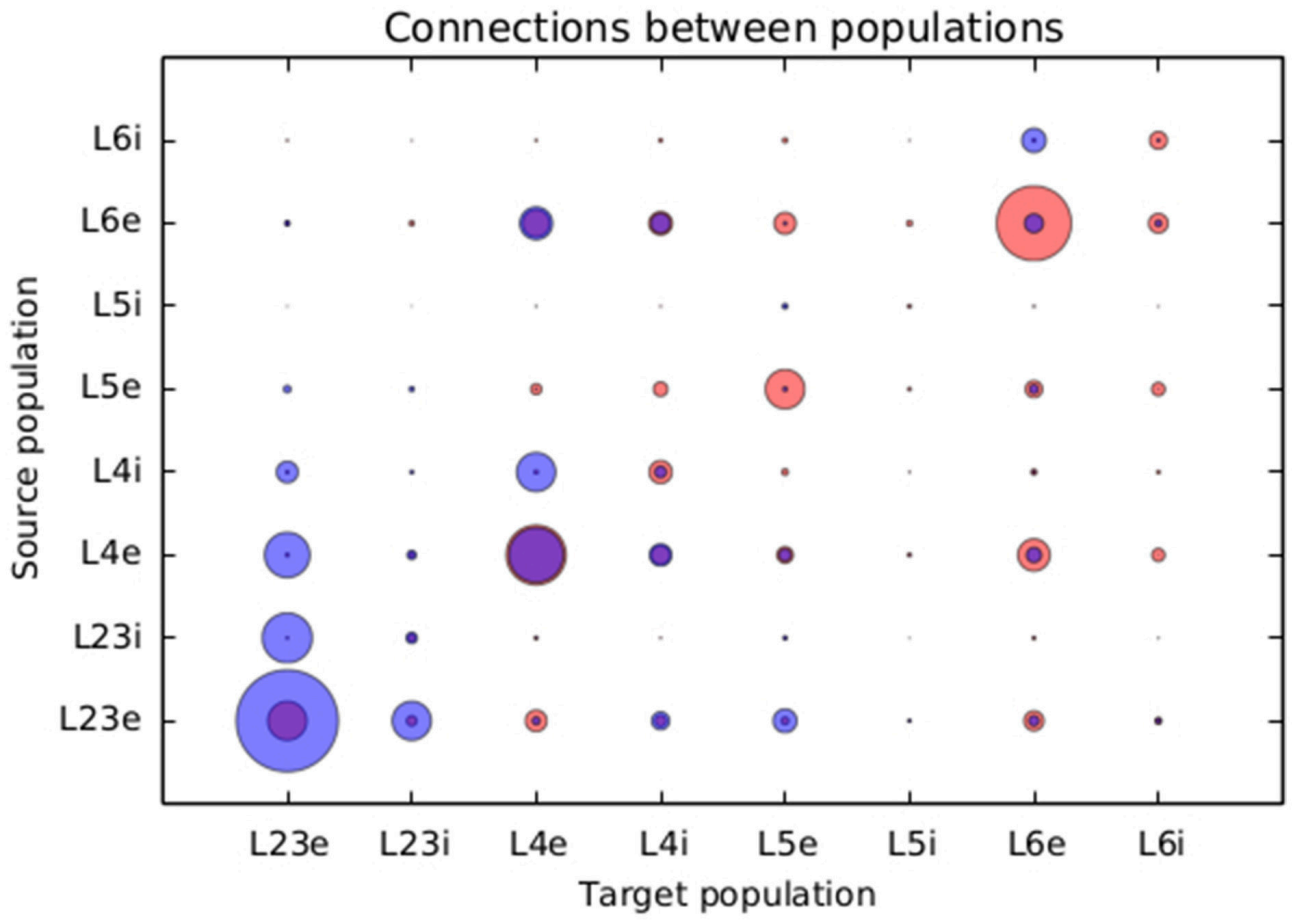
**Comparison of the normalized connectivity in the microcircuit model between the results obtained with the structural plasticity framework (red) and the values reported by Potjans and Diesmann (2014) (blue)**. The radius of the circle represents the linearly normalized value of the percentage of connections between layers.

Figure 8 presents a comparison between the proportional values of connectivity among layers between the results obtained from the simulation using structural plasticity and the original values reported by Potjans and Diesman. The average error in percentual connectivity is of 1.058 ± 1.175.

**Figure 9:**
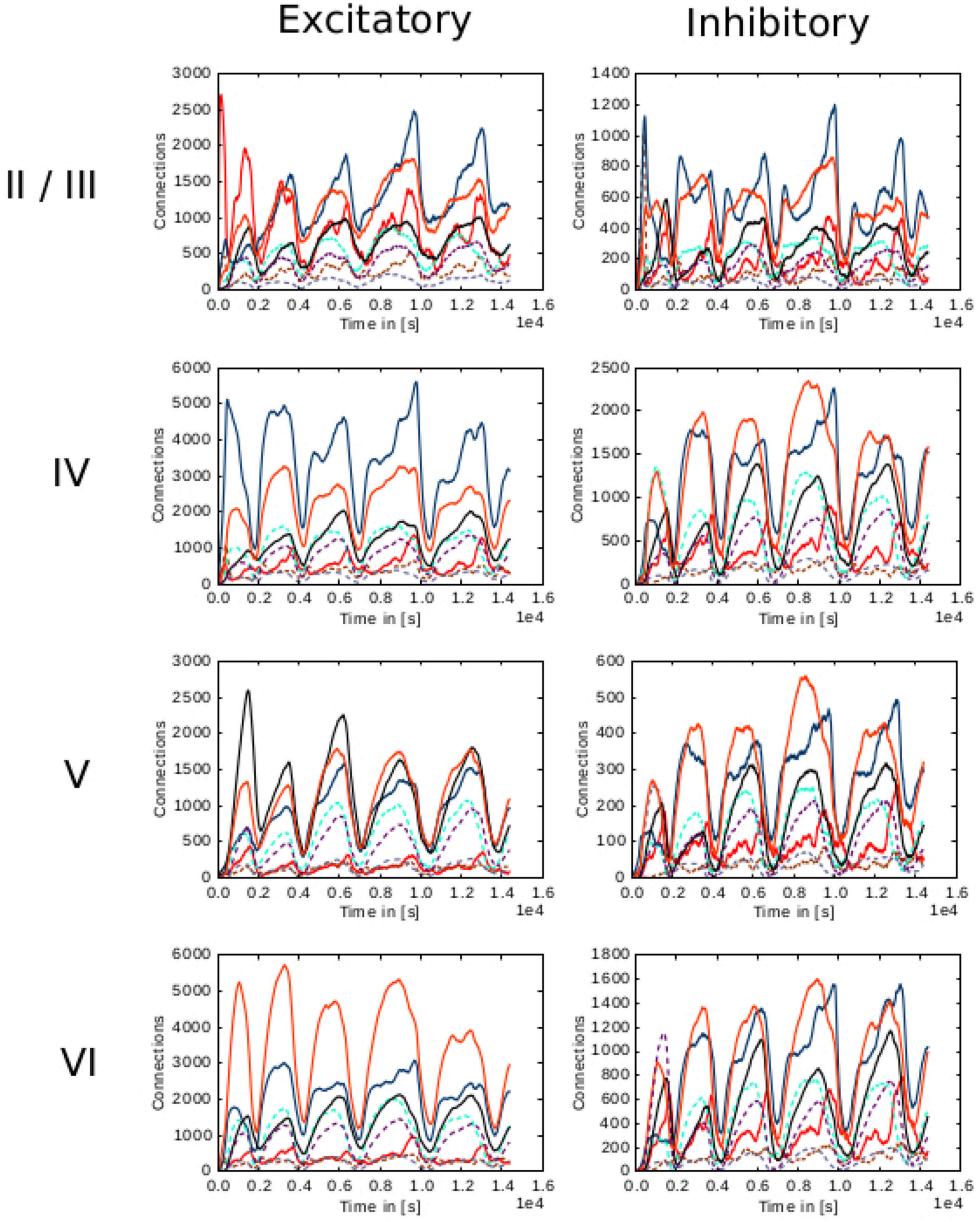
**Evolution of connectivity through time for each layer in the cortical microcircuit model with an unstable set of parameters**. Panels on the left illustrate connections incoming from excitatory populations. On the right, connections incoming from inhibitory populations. Rows show connections incoming from layers II/III, IV, V, and VI from top to bottom, respectively. On every panel, connections to excitatory populations in layers II/III (red), IV (blue), V (black), and VI (orange) are shown as solid curves, and connections to inhibitory populations in layers II/III (brown), IV (cyan), V (gray), and VI (purple) are shown as dashed curves.

**Figure 10:**
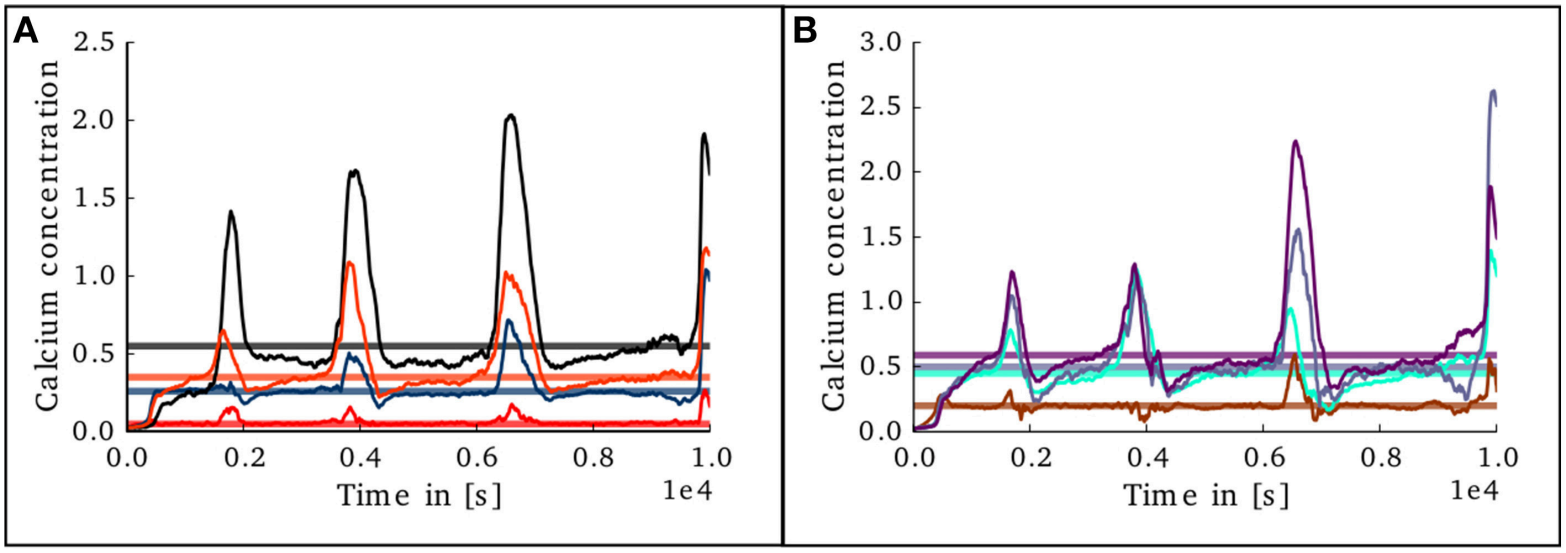
**Evolution of calcium concentration in each layer of the cortical microcircuit model with an unstable set of parameters**. **(A)** Shows the calcium concentration in the excitatory populations in layers II/III (red), IV (blue), V (black), and VI (orange). **(B)** Shows the calcium concentration in the inhibitory populations in layers II/III (brown), IV (cyan), V (gray), and VI (purple).

A second case was also explored, in which parameters lead to unstable network activity are chosen. Figure 9 shows the evolution of connectivity among layers and Figure 10 shows the changes in calcium concentration in each layer for this scenario. Overshoots in the connectivity, are originated by a choice of higher rate in the creation of synaptic elements. The system behaves as a feedback control system, with a delay which is defined by the time between updates in connectivity and the synaptic element creation rate. The synaptic element growth rate determines the steepness of the growth curve, and influences the speed at which control changes are made. The instability in the connectivity is reflected in the calcium concentration, never reaching the desired levels. A stable setting involves finding a suitable balance between the speed in the creation of excitatory and inhibitory connections related to the desired level of activity for each layer.

**Figure 11:**
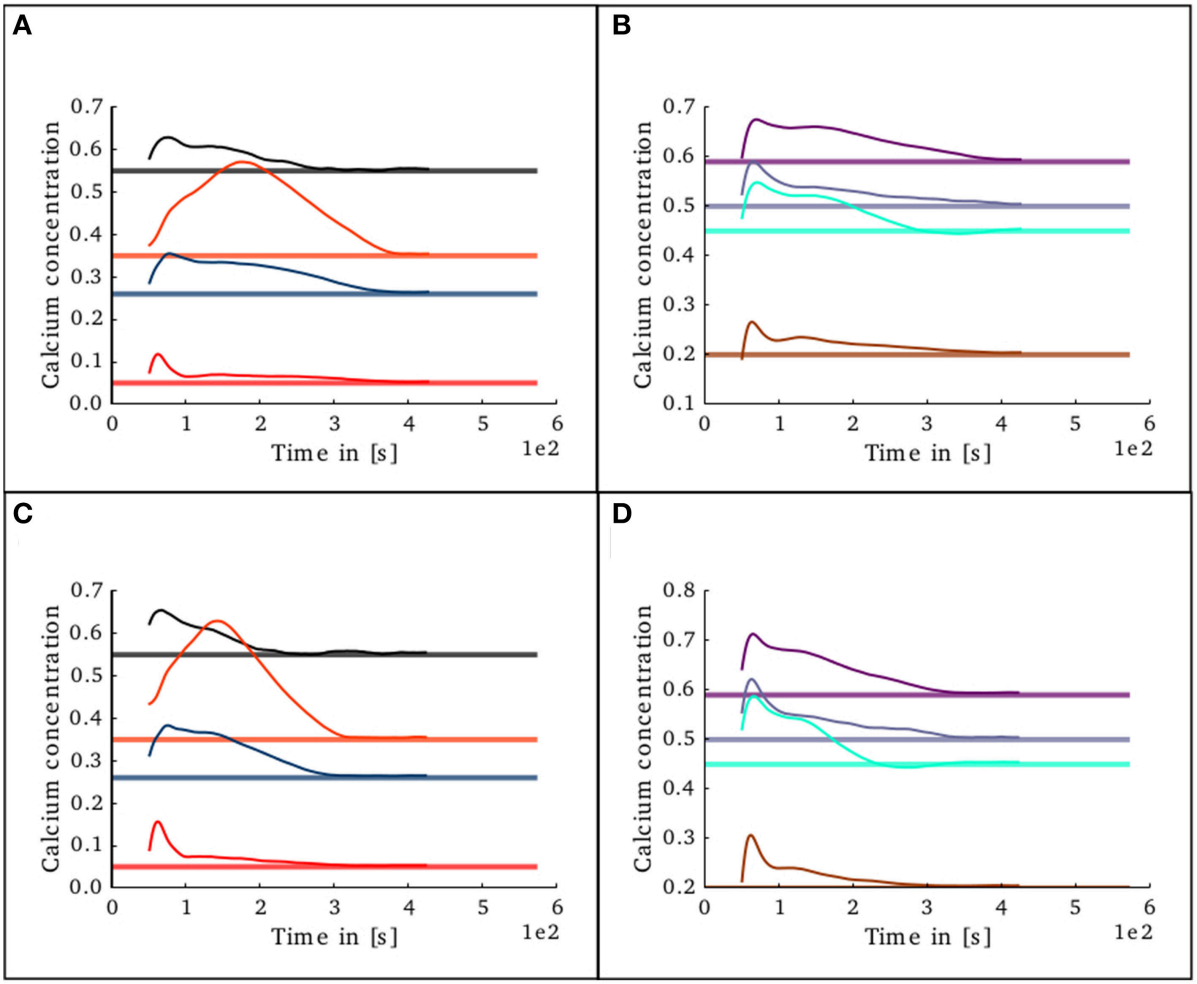
**Evolution of calcium concentration in each layer of the cortical microcircuit model with partially pre-connected initial conditions**. Pale horizontal lines indicate the target concentration of the corresponding population. Left pannels show excitatory populations in layers II/III (red), IV (blue), V (black), and VI (orange). Right pannels show inhibitory populations in layers II/III (brown), IV (cyan), V (gray), and VI (purple). **(A,B)** Show the scenario where the network was started with the connectivity as specified in the original work by Potjans and Diesman. **(C,D)** Show the same scenario but with a 10% error in the initial connectivity setup.

To study the behavior of the structural plasticity algorithm on partially pre-connected networks, another simulation was set in which the initial conditions in connectivity for the structural plasticity algorithm were those specified in the original model of Potjans and Diesman. The network was simulated without plasticity for an initial period of 30 s in order to allow the calcium concentration reach an initial stable value. The evolution of the calcium concentration in all layers after the structural plasticity algorithm was enabled can be seen in Figures 11A,B. The stability point is reached a lot faster than in the scenario with no initial connections, at around 400 s. A final simulation was set in which the connectivity was specified with a 10% error margin from the original setup reported by Potjans and Diesman. The evolution of the calcium concentration in all layers after plasticity was enabled can be seen in Figures 11C,D. The structural plasticity algorithm is able to find a suitable balance between excitation and inhibition. The initial overshoot in electrical activity is a reflection of the initial stronger reconfigurations of the network connectivity. It is important to highlight that a suitable growth scheme is required for the algorithm to reach this stability. Not all setups will become stable or find a solution, this depends on the initial conditions, the desired set points, the shape of the growth curve and the growth rate.

## 4. Discussion

In this paper we have described the implementation of a framework of structural plasticity for the neural network simulator NEST. We show that the framework is scalable and can be used to model the dynamical creation and deletion of synapses inside a large scale network guided by simple homeostatic rules.

This work also presents some use cases for the framework and some of its potential applications. Researchers can now use structural plasticity in NEST to generate the connectivity of a network from scratch, defining homeostatic rules, in form of synaptic element growth curves, which may vary according to their needs. The shape of the growth curve defines the speed with which new synaptic elements are created, and as a result, defines the acceleration at which calcium is stored inside the neuron. The relationship between the growth speed at certain level of calcium concentration of excitatory and inhibitory elements is fundamental to achieve stable setups under the model of structural plasticity. As is has been shown, some parameter combinations lead to unstable activity in the network. There are cases where the desired average electrical activity will never be reached by the system. In other cases the average electrical activity will oscillate continuously or suddenly go out of bounds. This relationship depends also on the size of the network and the neuron model used. As a consequence, some care is required in navigating the parameter space in order to achieve desired results.

The example of the two population network illustrates how this framework can be used to understand the interaction between activity and the creation of synapses. The behavior observed in the simulation can be used to model how inhibition triggers critical periods of connectivity during development of neural networks (Hensch, 2005). During this window, external stimuli can also be used to shape the formation of the new connections. Together with the performance measurements, these results show that our implementation of structural plasticity is suitable to study the development of connectivity patterns inside a neural network in an efficient and scalable manner.

In the specific case of the cortical microcircuit presented in this work, we are able to see some similarities and differences between the results obtained by simulating with the structural plasticity framework and the data reported in Potjans and Diesmann (2014). One of the most visible differences is the smaller amount of recurrent connections generated in the simulation for layer 2/3. This layer has a very low target electrical activity, which is initially almost reached by external input. This means that very few synapses are required to reach this target. This fact limits the creation of synapses for this layer. Note that the results shown in this paper were obtained only by defining target activity levels; no other connectivity constraints were specified. A more elaborate simulation could incorporate tailored growth curves for each layer, and implement additional connectivity restrictions which promote recurrent connections and other connectivity patterns that do not emerge naturally from the current approach.

Another visible difference is that the excitatory population of layers 5 and 6 show a higher number of connections than the ones shown in the original work. On the other hand, connections from and to the inhibitory population of layer 5 and layer 2/3 are well fit. Except from connections between the inhibitory and excitatory populations of layer 4, connections from and to layer 4 are also well predicted.

In this paper we describe a framework which can be used to study structural network dynamics. The focus of this paper is on the technical implementation. It is not the scope of the present work to perform a deep analysis of the biological results that can be obtained using this framework. However, we provide some examples of how the framework can be used, its capacities and limitations. This implementation gives researchers flexibility to explore complex connectivity dynamics by extending the synaptic elements growth rules. As our implementation is integrated into NEST, simulations using structural plasticity can also be combined with other features available in the simulator. For example, the user may take into account dynamic synaptic weights by mixing this framework with synaptic plasticity. The framework can also be further extended using the current topology framework in NEST in order to constrain connectivity by relative position.

We also show that the structural plasticity algorithm is able to solve the complex balance of interaction between layers with different levels of electrical activity when partial information of the connectivity is available. This result is very promising, as it shows that given the right growth rules, it would now be possible to reconstruct connectivity inside a network without having exact anatomical information. We therefore conclude that our approach represents a novel and useful technique to close the current gaps in information about the connectivity in certain regions of the brain.

## Author contributions

SD worked on the implementation of the structural plasticity framework in NEST, simulated the use cases and performed the scalability tests. MN did most of the implementation of the structural plasticity framework in NEST and assessed the simulations. MB gave the theoretical guidance regarding the structural plasticity algorithm and the use cases. AM gave scientific and theoretical guidance on the simulation and implementation of the framework.

## Funding

This work was supported by the Helmholtz Association through the Helmholtz Portfolio Theme “Supercomputing and Modeling for the Human Brain” and its Initiative and Networking Fund, and by the Jülich Aachen Research Alliance (JARA).

### Conflict of interest statement

The authors declare that the research was conducted in the absence of any commercial or financial relationships that could be construed as a potential conflict of interest.
