# Supplementary material for: Automatic Generation of Connectivity for Large-Scale Neuronal Network Models through Structural Plasticity
Source: Front Neuroanat. 2016 May 26;10:57. doi: 10.3389/fnana.2016.00057 (PMC4880596; doi:10.3389/fnana.2016.00057)
Supplement: Supplementary file 1 [file DataSheet1.PDF]

Aditional material: Example of a two population model using structural plasticity

```
import nest
import numpy
import matplotlib.pyplot as pl
class StructralPlasticityExample:
  def __init__(self):
      # General simulation parameters
      # simulated time (ms)
      self.t_sim = 150000.0
      # simulation step (ms).
      self.dt = 0.1
      self.number_excitatory_neurons = 800
      self.number_inhibitory_neurons = 200
      # Structural_plasticity and synapse properties
      self.update_interval = 100
      self.record_interval = 1000.0
      # rate of background Poisson input
      self.bg_rate = 10000.0
      self.neuron_model = 'iaf_psc_exp'
      # Growth curves for synaptic elements of excitatory neurons
      # Excitatory synaptic elements
      self.growth_curve_e_e = {
          'growth_curve': "gaussian",
          'growth_rate': 0.0001,
          'continuous': False,
          'eta': 0.0,
          'eps': 0.05,
      }
      # Inhibitory synaptic elements
      self.growth_curve_e_i = {
          'growth_curve': "gaussian",
          'growth_rate': 0.0001,
          'continuous': False,
          'eta': 0.0,
          'eps': self.growth_curve_e_e['eps'],
      }
      # Growth curves for synaptic elements of inhibitory neurons
      # Excitatory synaptic elements
      self.growth_curve_i_e = {
          'growth_curve': "gaussian",
          'growth_rate': 0.0004,
          'continuous': False,
          'eta': 0.0,
```

```
            'eps': 0.2,
        }
        # Inhibitory synaptic elements
        self.growth_curve_i_i = {
            'growth_curve': "gaussian",
            'growth_rate': 0.0001,
            'continuous': False,
            'eta': 0.0,
            'eps': self.growth_curve_i_e['eps']
        }
        # Neuron model
        self.model_params = {'tau_m': 10.0,   # membrane time constant (ms)
            'tau_syn_ex': 0.5,          # excitatory synaptic time constant (ms)
            'tau_syn_in': 0.5,          # inhibitory synaptic time constant (ms)
            't_ref': 2.0,               # absolute refractory period (ms)
            'E_L': -65.0,               # resting membrane potential (mV)
            'V_th': -50.0,              # spike threshold (mV)
            'C_m': 250.0,               # membrane capacitance (pF)
            'V_reset': -65.0,           # reset potential (mV)
        }
        self.nodes = ()
        self.nodes_i = ()
        self.spike_detector = ()
        self.data_ca_e = []
        self.data_ca_i = []
        # Initialize variables
        self.psc_e = 585.389956861
        self.psc_i = -585.389956861
        self.psc_ext = 6.20513354273
    def prepare_simulation(self):
        nest.ResetKernel()
        # set global kernel parameters
        nest.SetKernelStatus(
            {
                'resolution': self.dt
            }
        )
        # Set Structural Plasticity synaptic update interval
        nest.SetStructuralPlasticityStatus({
                'structural_plasticity_update_interval':
                self.update_interval,
            })
        # Define Structural Plasticity synapses
        nest.CopyModel('static_synapse', 'synapse_ex')
        nest.SetDefaults('synapse_ex', {'weight': self.psc_e, 'delay': 1.0})
        nest.CopyModel('static_synapse', 'synapse_in')
```

```
    nest.SetDefaults('synapse_in', {'weight': self.psc_i, 'delay': 1.0})
    nest.SetStructuralPlasticityStatus({
        'structural_plasticity_synapses': {
            'synapse_ex': {
                'model': 'synapse_ex',
                'post_synaptic_element': 'Den_ex',
                'pre_synaptic_element': 'Axon_ex',
            },
            'synapse_in': {
                'model': 'synapse_in',
                'post_synaptic_element': 'Den_in',
                'pre_synaptic_element': 'Axon_in',
            },
        }
    })

def create_nodes(self):
    # Assign the growth curves to the corresponding synaptic elements
    synaptic_elements = {
        'Den_ex': self.growth_curve_e_e,
        'Den_in': self.growth_curve_e_i,
        'Axon_ex': self.growth_curve_e_e,
    }
    synaptic_elements_i = {
        'Den_ex': self.growth_curve_i_e,
        'Den_in': self.growth_curve_i_i,
        'Axon_in': self.growth_curve_i_i,
    }
    #Create a population with 80% excitatory neurons
    self.nodes = nest.Create('iaf_neuron',
            self.number_excitatory_neurons, {
            'synaptic_elements': synaptic_elements
        })
    #Create a population with 20% inhibitory neurons
    self.nodes_i = nest.Create('iaf_neuron',
            self.number_inhibitory_neurons, {
            'synaptic_elements': synaptic_elements_i
        })
    nest.SetStatus(self.nodes, 'synaptic_elements', synaptic_elements)
    nest.SetStatus(self.nodes_i, 'synaptic_elements', synaptic_elements_i)

def connect_external_input(self):
    # Create and Connect the Poisson generator
    noise = nest.Create('poisson_generator')
    nest.SetStatus(noise, {"rate": self.bg_rate})
    nest.Connect(noise, self.nodes, 'all_to_all',
```

```
                {'weight': self.psc_ext, 'delay': 1.0})
        nest.Connect(noise, self.nodes_i, 'all_to_all',
                {'weight': self.psc_ext, 'delay': 1.0})
        self.spike_detector = nest.Create('spike_detector')
        nest.Connect(self.nodes, self.spike_detector, 'all_to_all')
        nest.Connect(self.nodes_i, self.spike_detector, 'all_to_all')

  def record_ca(self):
        t = nest.GetKernelStatus('time')
        buffer = nest.GetStatus(self.nodes, 'Ca'),
        self.data_ca_e = self.data_ca_e + [numpy.mean(buffer)]
        buffer = nest.GetStatus(self.nodes_i, 'Ca'),
        self.data_ca_i = self.data_ca_i + [numpy.mean(buffer)]

  def plot_data(self):
        pl.figure()
        pl.axhline(self.growth_curve_e_e['eps'],linewidth=4.0, color='#9999FF')
        pl.plot(self.data_ca_e,'b',
               label='Ca Concentration Excitatory Neurons',linewidth=2.0)
        pl.axhline(self.growth_curve_i_e['eps'],linewidth=4.0, color='#FF9999')
        pl.plot(self.data_ca_i,'r',
               label='Ca Concentration Inhibitory Neurons',linewidth=2.0)
        pl.xlabel("Time in [s]")
        pl.ylabel("Ca concentration")

  def simulate(self):
        nest.EnableStructuralPlasticity()
        sim_steps = numpy.arange(0, self.t_sim, self.record_interval)
        for i, step in enumerate(sim_steps):
            nest.Simulate(self.record_interval)
            self.record_ca()
        self.plot_data()

if __name__ == '__main__':
  example = StructralPlasticityExample()
  # Prepare simulation
  example.prepare_simulation()
  example.create_nodes()
  example.connect_external_input()
  # Start simulation
  example.simulate()
```
