# Supplementary material for: Automatic Generation of Connectivity for Large-Scale Neuronal Network Models through Structural Plasticity
Source: Front Neuroanat. 2016 May 26;10:57. doi: 10.3389/fnana.2016.00057 (PMC4880596; doi:10.3389/fnana.2016.00057)
Supplement: Supplementary file 2 [file DataSheet2.pdf]

Apendix A: Stable and unstable parameter setting for the cortical microcircuit model

**Parameter**	**Stable case**	**Unstable case**
Growth curve type	Gaussian	Gaussian
Growth rate excitatory dendritic	0.0001	0.0001
Growth rate excitatory axonal	0.00018	0.0001
Growth rate inhibitory dendritic	0.0001	0.0001
Growth rate inhibitory axonal	0.00025	0.0001
η excitatory	0.0	0.0
η inhibitory	0.0	0.0
ϵ excitatory L23 dendritic	0.05	0.05
ϵ inhibitory L23 dendritic	0.2	0.2
ϵ excitatory L23 axonal	0.05	0.05
ϵ inhibitory L23 axonal	0.2	0.2
ϵ excitatory L4 dendritic	0.26	0.26
ϵ inhibitory L4 dendritic	0.45	0.45
ϵ excitatory L4 axonal	0.26	0.26
ϵ inhibitory L4 axonal	0.45	0.45
ϵ excitatory L5 dendritic	0.55	0.55
ϵ inhibitory L5 dendritic	0.5	0.5
ϵ excitatory L5 axonal	0.55	0.55
ϵ inhibitory L5 axonal	0.5	0.5
ϵ excitatory L6 dendritic	0.35	0.35
ϵ inhibitory L6 dendritic	0.59	0.59
ϵ excitatory L6 axonal	0.35	0.35
ϵ inhibitory L6 axonal	0.59	0.59

Table 1: Parameters used for simulations of the cortical microcircuit
